# Bioprinting of Adult Dorsal Root Ganglion (DRG) Neurons Using Laser-Induced Side Transfer (LIST)

**DOI:** 10.3390/mi12080865

**Published:** 2021-07-23

**Authors:** Katiane Roversi, Hamid Ebrahimi Orimi, Marcelo Falchetti, Edroaldo Lummertz da Rocha, Sebastien Talbot, Christos Boutopoulos

**Affiliations:** 1 Département de Pharmacologie et Physiologie, Université de Montréal, Montréal, QC H3C 3J7, Canada; katiane.roversi@umontreal.ca (K.R.); sebastien.talbot@umontreal.ca (S.T.); 2 Centre de Recherche Hôpital Maisonneuve-Rosemont, Montréal, QC H1T 2M4, Canada; h.e.orimi@hotmail.com; 3 Department of Mechanical, Industrial and Aerospace Engineering, Concordia University, Montréal, QC H3G 1M8, Canada; 4 Department of Microbiology, Immunology and Parasitology, Federal University of Santa Catarina, Florianópolis 88040-900, Brazil; marcelofalchetti09@gmail.com (M.F.); edroaldo.lummertz@ufsc.br (E.L.d.R.); 5 Département d’Ophtalmologie, Université de Montréal, Montréal, QC H3C 3J7, Canada; 6 Institut de Génie Biomédical, Université de Montréal, Montréal, QC H3C 3J7, Canada

**Keywords:** laser-induced side transfer, laser-assisted bioprinting, adult DRG neurons, sensory neurons, viability, calcium kinetics, transcriptome

## Abstract

Cell bioprinting technologies aim to fabricate tissuelike constructs by delivering biomaterials layer-by-layer. Bioprinted constructs can reduce the use of animals in drug development and hold promise for addressing the shortage of organs for transplants. Here, we sought to validate the feasibility of bioprinting primary adult sensory neurons using a newly developed laser-assisted cell bioprinting technology, known as Laser-Induced Side Transfer (LIST). We used dorsal root ganglion neurons (DRG; cell bodies of somatosensory neurons) to prepare our bioink. DRG-laden- droplets were printed on fibrin-coated coverslips and their viability, calcium kinetics, neuropeptides release, and neurite outgrowth were measured. The transcriptome of the neurons was sequenced. We found that LIST-printed neurons maintain high viability (Printed: 86%, Control: 87% on average) and their capacity to release neuropeptides (Printed CGRP: 130 pg/mL, Control CGRP: 146 pg/mL). In addition, LIST-printed neurons do not show differences in the expressed genes compared to control neurons. However, in printed neurons, we found compromised neurite outgrowth and lower sensitivity to the ligand of the TRPV1 channel, capsaicin. In conclusion, LIST-printed neurons maintain high viability and marginal functionality losses. Overall, this work paves the way for bioprinting functional 2D neuron assays.

## 1. Introduction

Three-dimensional cell bioprinting technologies enable precise delivery and positioning of cells and incorporating extracellular components for the fabrication of complex living constructs [1]. They find applications in the development of efficient drug screening models, in vitro modelling, as well as in the generation of tissue and organs for transplantation. Recently, 3D printing technologies have been combined with biomaterials and compounds that change in a dynamic way (i.e., self-assembly, drug release). Printed constructs can mimic organ responses and/or interact with them. These developments are part of the rapidly growing field of multidimensional printing [2,3].

Bioprinting technologies can be categorized into four main categories, namely: material jetting (e.g., ink-jet printing [4], laser-induced forward transfer (LIFT)); vat photopolymerization (e.g., stereolithography); pneumatic or mechanical material extrusion [5]; and free-form spatial printing [6]. Depending on the printing mechanism, these technologies present partial compatibility with available bioink formulations, with the bioink viscosity being the limiting factor [7]. For instance, the bioink viscosity printability range in ink-jet printing is 3.5–12 mPa·s, while the corresponding range for microextrusion is 30 mPa·s to > 6 × 10^7^ mPa·s. Laser-assisted bioprinting can support a wide range of bioink viscosities with marginal effects on cell viability and function [8,9,10], while having the advantages of high printing resolution and reproducibility [8,11]. Laser-induced forward transfer (LIFT) is the most common laser-assisted bioprinting technology. LIFT uses focused laser pulses to propel bioink drops from a donor substrate onto a collector substrate, achieving high printing resolution and cell density [12]. By using a nozzleless approach, LIFT is compatible with bioinks having a wide viscosity range (1–300 mPa·s). However, 3D printing capabilities remain limited in LIFT due to unresolved donor preparation challenges [13]. A modified methodology for the laser bioprinting of cells, called Laser Induced Side Transfer (LIST), was recently developed by our group [13]. LIST uses low energy nanosecond laser pulses to generate a transient microbubble at the distal end of a glass microcapillary supplied with bioink. Microbubble expansion results in the ejection of a cell-laden microjet perpendicular to the irradiation axis. We previously showed that LIST-printed human umbilical vein endothelial cells (HUVECs) present negligible loss of viability, and maintain their abilities to migrate, proliferate and form intercellular junctions [13]. This method is technically uncomplicated and aims to cover a technological gap in the drop-on-demand bioprinting field: the lack of technologies that can 3D print large-scale constructs using both high and low viscosity bioinks. Although similar technology [14] has shown compatibility with a wide bioink viscosity range (2–200 mPa·s), this is yet to be tested in cell-laden bioinks delivered by LIST.

Three-dimensional bioprinting was recently used for neural tissue engineering as a platform to mimic the mechanical, structural, and cellular properties of central and peripheral nervous system tissues. The emergence of neuronal bioprinted platforms can facilitate disease modeling and drug screening applications, as well as the fabrication of implants for in vivo regenerative therapies within the central and peripheral nervous systems [15]. Embryonic neuronal cell types, including hippocampal, cortical and motor neurons, were previously printed using ink-jet printing and tested for post-printing functionality [16,17]. LIFT has already shown the potential to print primary dorsal root ganglion (DRG) neurons [18]. However, bioprinting of the adult neuronal cells of the central nervous system (CNS) or peripheral nervous system (PNS) has been less explored—presumably, due to the limited ability of those cells to survive thermomechanical stress and regenerate [19].

Here, we sought to validate whether LIST is compatible with primary DRG neurons. We will present a comprehensive characterization of LIST-printed DRG neurons, including comparative results on viability, neurite outgrowth, sensitivity to noxious stimuli, ability to release neuropeptides, and transcriptome.

## 2. Materials and Methods

Neurons. The neurons were derived from dorsal root ganglions (DRG) of C57BL/6J (#000664, Jackson Laboratory, Bar Harbor, MA, USA) and VGlut2^cre^:td-tomato^fl/wt^ of 6 to 8 weeks of age. For the ganglion extraction, the mice were euthanized and the DRGs harvested out into DMEM medium (completed with 50 U/mL penicillin and 50 μg/mL streptomycin (#MT-3001-Cl, Fisher, Waltham, MA, USA), 2 mM L-Glutamine (#25030-081, Life Technologies), and 10% Hi FBS (#10082-145, Life Technologies, Pasir Ris, Singapore). For the cell’s dissociation, the DRGs were incubated within HEPES buffered saline (#51558, Sigma, Burlington, MA, USA) completed with 1 mg/mL collagenase A (#1108879300, Sigma) + 2.4 U/mL dispase II (#4942078001, Sigma) for 80 min at 37 °C. The ganglions were triturated with glass Pasteur pipettes in DMEM medium + DNAse (#EN052, ThermoScientific, Waltham, MA, USA), then centrifuged over a 15% BSA (#SH30574.02, HyClone/Fisher Scientific, Waltham, MA, USA) gradient in PBS, washed and then resuspended in Neurobasal-A medium with 0.05 ng/μL NGF (#13257-019, Life Technologies), 0.002 ng/μL GDNF (#450-51-10, Peprotech, Rocky Hill, CA, USA) and 0.01 mM AraC (#C6645, Sigma).

Bioink and printing substrate preparation. The bioink was prepared using 10^6^ DRG neurons per ml suspended in Neurobasal-A medium with NGF, GDNF, and AraC, fibrinogen (13.24 µM) (F8630-5G; Sigma-Aldrich, Burlington, MA, USA) and Allura red AC (458848-100G, Sigma-Aldrich), (10 mM) as a light absorber. The printing substrates were fibrin-coated 18 mm microscope round cover glasses (48380-046, VWR, Tamil Nadu, India). For the fibrin gel coating (~1 mm-thick), we used 242 μL of a Basal medium (SCME001, Millipore), containing fibrinogen (13.24 μM) (F8630-1G, Sigma) and 8 μL of a thrombin solution (3.2 U/mL final concentration in the fibrin gel) (T7513- 100UN, Sigma-Aldrich). We used drop-casting to deposit the two solutions onto the microscope cover glasses one hour before printing.

Printing protocol. Freshly prepared bioink (~100 μL) was loaded onto a squared capillary (Vitrocom hollow square capillary, inner size 0.3 mm × 0.3 mm, 0.15 mm wall thickness, 50-mm long) using a syringe pump (NE-1000, New Era Pump Systems Inc., Farmingdale, NY, USA). The laser beam was focused on the middle of the capillary, 500 μm away from its distal end, using a 4× objective lens (plan achromat, NA = 0.1, Olympus, Tokyo, Japan). The receiving substrate was fixed on an XYZ translation stage and placed 500–700 μm away from the capillary tip. Printing laser energies, measured at the sample, were 100 or 120 μJ. Printing patterns consisted of arrays of individual droplets separated by a 500 μm gap. A detailed description of the printing setup can be found in our previous work [13]. After printing, the samples were placed in an incubator for 20 min, then rinsed twice with Neurobasal-A medium, completed with 2 mL of neurobasal-A medium, and put back in the incubator for 48 h.

Viability assay. Neuron viability was determined with fixable Viability Dye (VD) eFluor™ 780 (#65-0865-14, Invitrogen, Waltham, MA, USA) staining. After 48 h in culture, the coverslips containing the cells were incubated with the VD dye (1:1000 dilution) in neurobasal for 1 h in an incubator, then washed 3 times with PBS, fixed with 10% Neutral buffered formalin for 10 min, washed 3 times with PBS, and mounted with a rectangular coverslip using Fluoromount-G™ Mounting Medium, with DAPI (#00-4959-52, Invitrogen). Fluorescence images were captured using a Zeiss AxioImager Z2 microscope coupled to an AxioCam MRc color CCD camera. The images were analyzed using a MATLAB algorithm which detects the nuclei of all printed cells (blue-DAPI stained), the marker of neurons (Red-td-tomato), and the cells stained or not by the viability dye (purple).

Neurite outgrowth. For the quantification of the neurite length of each neuron, we used the *NeuronJ* plug-in of NIH-ImageJ software (version 2.1.0). For this, 8-bit grayscale images of fluorescent neurons with identifiable neurites were loaded into the software and calibrated according to the image magnification. The average length of the neurites was obtained by manually tracing the length of all neurite outgrowths from the neuron’s cell body, divided by the total number of neurites per neuron. Neurite lengths and total number of neurites were averaged across all neurons in each glass coverslip and plotted.

CGRP release assay. For the CGRP release assay, the neurons were cultured for 48 h, then exposed to 1 uM capsaicin (#0462, Tocris, Bristol, UK) or vehicle for 10 min at 37 °C. The supernatants were collected and the CGRP was measured using the Rat CGRP Enzyme Immunoassay Kit (#589001, Bertin Pharma/Cayman Chemical, Bristol, UK). Plates were read at 414 nm on a Synergy H1 microplate reader (#19121628, Biotek, Orleans, VT, USA) [20].

Calcium imaging. After 48 h in culture, neurons were loaded with 5 μM Fura-2 AM (#2243-1, Biovision, Milpitas, CA, USA) at 37 °C for 45 min in Neurobasal-A medium, then washed with Standard Extracellular Solution (SES, 145 mM NaCl, 5 mM KCl, 2 mM CaCl_2_, 1 mM MgCl2, 10 mM glucose, 10 mM HEPES, pH 7.5). Their response to the noxious ligand 100 nM capsaicin (TRPV1 agonist) and 40 mM of KCL (positive control) was analyzed at room temperature. The ligands were dispersed (30 s) onto neurons using perfusion barrels followed by buffer washout of 210 s. For the imaging acquisition, the neurons were illuminated with a UV light source (Xenon lamp, 75 watts, Nikon, Melville, NY, USA), 340 nm and 380 nm excitation alternated by an LEP MAC 5000 filter wheel (Spectra services), and fluorescence emission captured by Cool SNAP ES camera (Princeton Instruments, New Jersey, MA, USA). We processed, background corrected and analyzed 340/380 ratiometric images (IPLab v2.8.0 software) and Microsoft Excel was used for post hoc analyses.

RNA sequencing. Neurons were stored in Trizol in −80 °C until use. The RNA was extracted using the PureLink RNA Micro Kit (Invitrogen, #12183-016). RNA was purified and subjected to TruSeq stranded mRNA library preparation for mouse according to the manufacturer’s instructions (Illumina, San Diego, CA, USA). Quality control was performed for RNA extraction and cDNA library preparation steps. The libraries were sequenced on an Illumina NovaSeq 6000 sequencing platform, yielding at least 25 million reads per sample. mRNA library preparation and sequencing were performed at Genome Quebec facilities. The reads were aligned using STAR (Spliced Transcripts Alignment to a Reference) to mouse reference genome (GRCm38, release 83), sorted the bam files by names using SAMtools and counted reads using featureCounts.

The differential expression analysis between the groups “Printed cells” and “Control cells” were performed in R environment and a non-specific filtering of genes with 0 counts in the six samples and of genes with the 25% lowest variance values between samples. Size factors, estimates for Negative Binomial distributed data were estimated and tested the significance of coefficients in a Negative Binomial Generalized Linear Models (GLM) using the DESeq2 package. Were considered as differentially expressed genes those that presented absolute log2 Fold Change > 1 and adjusted (Benjamini and Hochberg method) *p*-value < 0.05. Gene symbols were obtained using the biomaRt package. The volcano plot representation was built using the ggplot2 package. The Pearson correlation between the samples regularized transcriptional profile was obtained using the cor package and visualized it using the pheatmap package with Euclidean distance between the samples and “ward.D2” agglomeration method. These data have been deposited in the National Center for Biotechnology Information (NCBI)’s Gene Expression Omnibus (accession number pending) and are accessible for download at http://www.talbotlab.com (accessed date: 22 July 2021).

Statistical analysis. Results are expressed as mean ± standard error of the mean (S.E.M.) in all experiments. The statistical significance was tested by one-way ANOVA with post hoc Tukey or two-tailed unpaired Student T-test for single comparison. Values were considered significantly different when *p* < 0.05. Statistical computations and graphs were made with GraphPad Prism software (version 9.0.2).

## 3. Results and Discussion

### 3.1. Effects of the Printing Process on DRG Neurons Survival and Neurite Outgrowth

**Figure 1:**
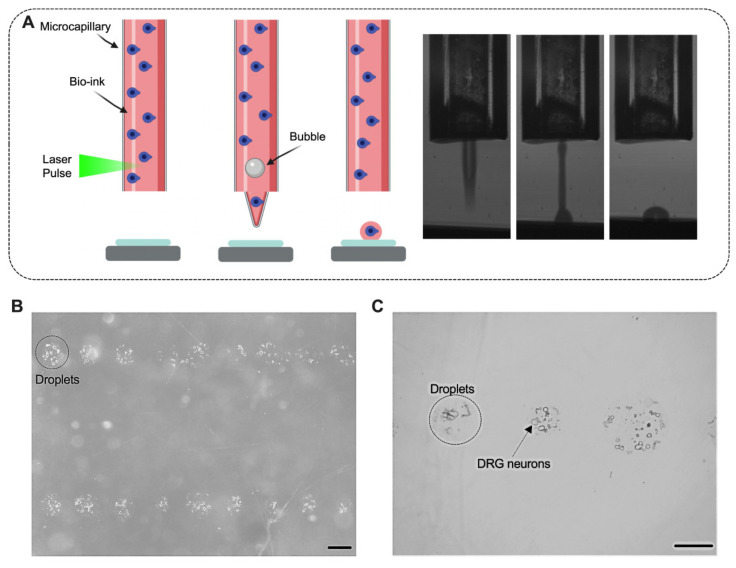
Laser-induced side transfer (LIST) of neurons. Schematic representation of the printing system (**A** left) and indicative high-speed imaging of bioink ejection (**A** right). Printed droplets with DRG neurons 1 h after printing. (**B**,**C**). Scale bar = 50 µM (**B**,**C**).

**Figure 2:**
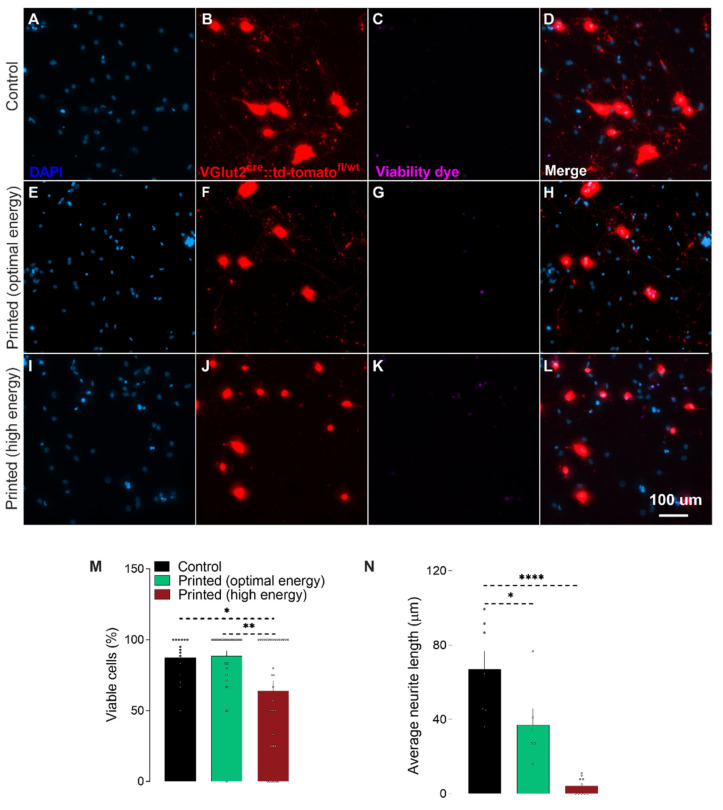
Bioprinting does not impact DRG neurons’ survival, but reduces neurite outgrowth. Representative fluorescence images of adult VGlut2^cre^::td-tomato^fl/wt^ DRG neurons mice printed with low (100 µJ, **E**–**H**) or high energy (120 µJ, **I**–**L**) or put in cell culture (control, **A**–**D**). Percentage of viable cells 2 days post-printing (**M**; determined as fixable Viability Dye eFluor™ 780^-^) and average neurite length (**N**, in µm). Results consist of the means ± S.E.M. One-way ANOVA with Tukey’s multi comparisons test. Significant differences in **M** and **N** are indicated by asterisks (*p* < 0.05 = *; *p* < 0.01 = **; *p* < 0.0001 = ****). Nucleus (Blue; **A**,**E**,**I**), VGlut2^cre^::td-tomato^fl/wt^ neurons (red; **B**,**F**,**J**), dead cells (purple; **C**,**G**,**K**). Scale bar = 50 µM (**A**–**L**).

We first sought to quantify potential effects of the printing process on the cell viability. For this, neuron-laden droplets were printed using two laser energies (100 and 120 μJ) (Figure 1). The selection of the laser energies was based on our previous work on LIST [13]. After printing, the cells were cultured for 2 days and the cell viability was assessed (Figure 2A–L). We found that LIST-printed neurons maintain high viability when printed at the optimal energy of 100 μJ (Printed: 86%, Control: 87% on average), while a decrease in the viability was found for printing at the higher laser energy of 120 μJ (64%) (Figure 2M). The higher cell death observed at 120 μJ may be due to increased thermomechanical impact on the cells upon exposure to higher energies. Orimi et al. reported marginal loss of viability in LIST-printed HUVECs using different laser energies (90–120 µJ) [13]. However, the viability of LIST-printed DRG neurons is considerably lower at high laser energy (120 µJ) because of the inherent sensitivity of primary DRG neurons. We then tested the ability of LIST-printed neurons to grow neurites. We found compromised neurite outgrowth in printed neurons for both optimal energy (44% less than the control group) and high energy (94% less than the control group) (Figure 2N).

Curley et al. have printed primary embryonic DRG neurons using LIFT. Although past reports have documented minimal effects of LIFT on cell viability, their study documented considerable viability loss in the printed group (84.9%) when compared to the control (95.6%) and to a cancer cell lineage printed under the same conditions (95.6%). The authors attributed the viability compromise to the high sensibility of this population of neurons. In an inkjet printing study, reduced neurite development and loss in viability after 5 days in culture was observed for retinal ganglion cells (RGC) [21]. The authors attributed the impairment in the neurite growth to the greater sensitivity of the adult cells in the culture, as well as the lower density and proximity of the neurons in the printed group. Interestingly, the authors found that coculturing those adult printed RGC neurons with glial cells was able to protect them from impaired viability and neurite outgrowth [21]. Therefore, in future studies, the addition of satellite glial cells could be explored as a means to improve the neurite growth of LIST-printed adult DRG neurons. Note that we found superior neuron viability in our study compared to piezoelectric inkjet printing [21]. This might be due to the lower liquid ejection speed in LIST (3.2 to 11.6 m/s [13]) compared to that used in inkjet printing (10 to 13 m/s [21]). Note that the higher the ejection speed, the higher the mechanical impact on the cells upon deposition. Overall, the cell viability comparison is consistent with the broader LIFT literature showing better cell viability compared to inkjet printing [7].

Taken together, these results indicate that LIST with the optimal laser energy (100 µJ) does not affect the viability of the neurons but limits their ability to extend neurites.

### 3.2. Effects of the Printing Process on Calcium Influx and Neuropeptide Release

**Figure 3:**
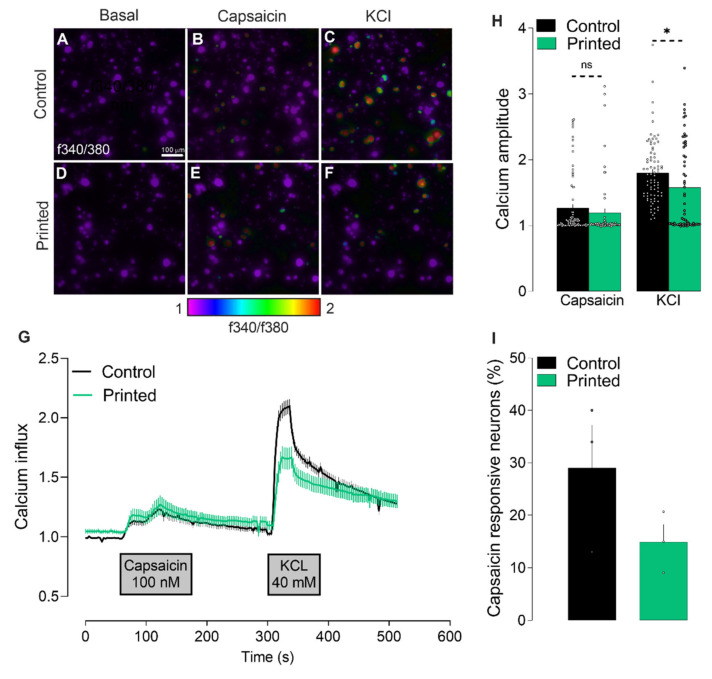
Effects of the printing process on calcium influx triggered by capsaicin. Calcium flux (**A**–**F**, showed as heatmap of f340/f380; (**G**), showed as time–response curve of f340/f380) in cultured (**A**–**C**) and printed (**D**–**F**) DRG neurons after exposure to vehicle (**A**,**D**), capsaicin (100 nM; **B**,**E**) and KCl (40 mM; **C**,**F**). The maximum amplitude of response evoked by capsaicin and KCl (**H**). Percentage of capsaicin-responsive neurons (**I**). Results consist of the means ± S.E.M. Two-tailed unpaired Student t-test. Significant differences are indicated by asterisks (*p* < 0.05 = *; ns: not significant as *p* > 0.05). Amplitude represents the point in which the change in the ratio f340/f380 is maximal. Scale bar = 100 µM (**A**–**F**).

We tested the ability of LIST-printed neurons to respond to ligands of ion channel receptors expressed on nociceptors. TRPV1 is such a receptor, specialized in noxious heat-sensing (~42–45 °C) and expressed by ~40% of nociceptors. Once activated, TRPV1 triggers the uptake of sodium and calcium (Ca^2+^) through its ionic pore, leading to neuron depolarization and neuropeptides’ release [22]. The presence and responsiveness of this channel can be measured by calcium microscopy. For this, DRG neurons were loaded with the fluorescent intracellular calcium indicator Fura-2 AM, exposed to the TRPV1 agonist capsaicin and potassium chloride (KCl), and calcium influxes were measured by monitoring changes in fluorescence. We found that the LIST-printed DRG neurons have a similar response to capsaicin, while they had a decrease in the response to KCl when compared to the control neurons (Figure 3G,H). In addition, we found a trend indicating that a lower percentage of neurons was activated by capsaicin, in comparison to control neurons (Figure 3I).

**Figure 4:**
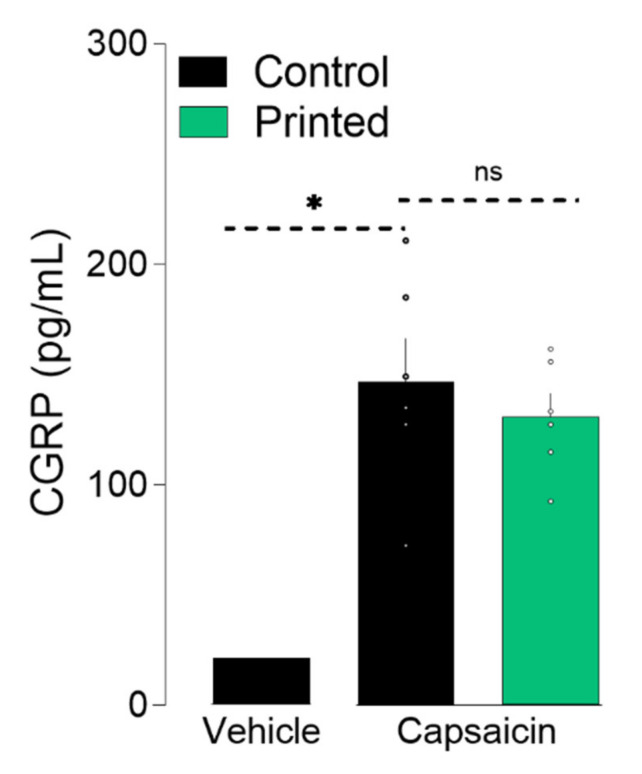
Printed-DRG neurons release neuropeptide. DRG neurons were printed and cultured for 48 h and exposed to capsaicin (1 uM). Supernatant was harvested 10 min after capsaicin exposure and CGRP release was measured by ELISA. Results consist of the means ± S.E.M. Individual values are represented with “◦”.One-way ANOVA with Tukey’s multi comparisons test. Significant differences are indicated by asterisks (*p* < 0.05 = *; ns: not significant as u > 0.05).

The electrochemical transmission of action potential requires the release of neuropeptides and neurotransmitters at the synapse. A sufficient increase in cytosolic Ca^2+^ concentration leads to neuropeptides’ release [23]. Thus, to further validate the integrity of LIST-printed DRG neurons, we measured calcitonin gene-related peptide (CGRP) release upon capsaicin exposure. To do so, we exposed the cultured and printed neurons to capsaicin for 10 min, harvested the supernatant, and measured the CGRP content using an enzyme-linked immunosorbent assay. We found that the LIST-printed DRG neurons maintained their ability to release CGRP (Printed: 130 pg/mL, Control: 146 pg/mL; Figure 4). Taken together, these results indicate that LIST-printed neurons: (i) expressed functional TRPV1 channels; (ii) that their TRPV1 sensitivity is not compromised; and (iii) that they maintain their ability to communicate with the cells of their environment by means of peptide release.

To the best of our knowledge, this is the first study measuring the calcium influx and neuropeptide release in bioprinted adult DRG neurons. Using 3D bioprinted iPSC-derived spinal neurons, Joung and collaborators [24] have shown a normal calcium influx in response to KCl and glutamate, which is evidence of active and functionally mature neurons. Other studies have measured neurons activity using whole-cell patch-clamp, a technique that records the electrophysiological properties of the neurons following the injection of current. Xu and collaborators have found similar electrophysiological behavior between control and thermally inkjet-printed embryonic primary hippocampal and cortical neurons [17]. On the other hand, Kador and collaborators found that inkjet-printed RGC neurons required higher current to develop the same response as control neurons [25]. These findings show that the functional activity of printed neurons varies according to the subtype of neurons, as well as the printing methodology used.

### 3.3. Effects of the Printing Process on the Expressed Genes

**Figure 5:**
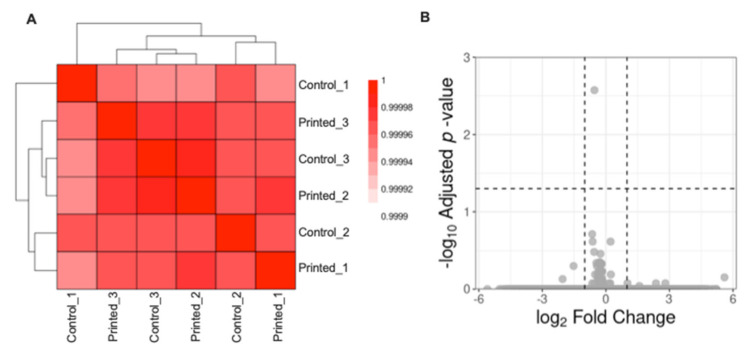
Printed nociceptor neurons transcriptome. Clustering of the samples showing the Pearson correlation value between each pair of samples (**A**). Volcano plot representation of differentially expressed genes between Control and Printed neurons (**B**).

Finally, we tested whether LIST-printed neurons show differentially expressed gene profiles. For this, we cultured control and LIST-printed DRG neurons for 2 days and then we collected the neurons, isolated the RNA and quantified the RNA using next-generation sequencing. We identified a strong correlation between the total transcriptional profile of control cells and LIST-printed DRG neurons (Pearson correlation > 0.99; Figure 5A) and we did not identify differentially expressed genes in this contrast using the established thresholds (absolute log_2_ fold change > 1; adjusted *p*-value < 0.05; Figure 5B). These results indicate that the printing process does not alter the transcriptome of the printed cells.

## 4. Conclusions

In conclusion, we show that LIST-printed adult sensory neurons maintain high viability and functional integrity. Overall, this work paves the way for bioprinting functional 3D sensory neuron assays and opens possibilities for developing bioprinted grafts for use in nerve recovering medicine.

## Data Availability

The data that support the findings of this study are available from the corresponding author upon reasonable request. Sequencing data are deposited on www.talbotlab.com (accessed date: 22 July 2021).
